# Supplementary material for: Genome-Wide Association Study of the Reproductive Traits of the Dazu Black Goat (Capra hircus) Using Whole-Genome Resequencing
Source: Genes (Basel). 2023 Oct 19;14(10):1960. doi: 10.3390/genes14101960 (PMC10606515; doi:10.3390/genes14101960)
Supplement: Supplementary file 1 [file genes-14-01960-s001.zip › genes-2640348-supplementary.pdf]

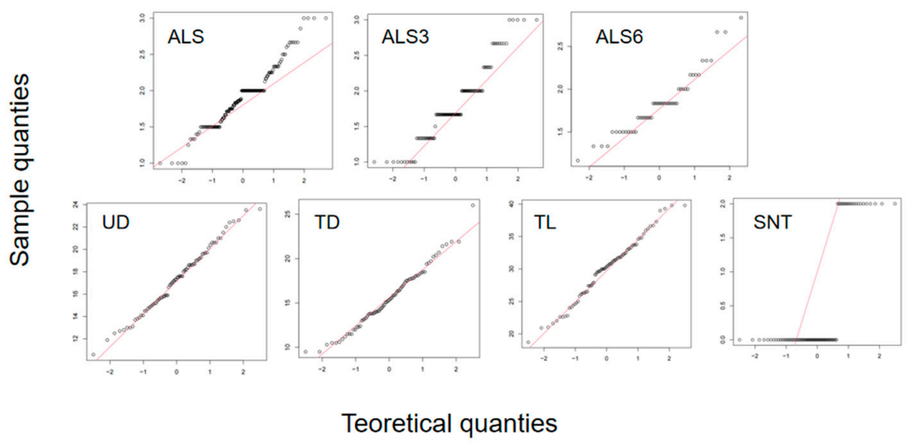

Figure S1. QQ Diagram for normality Test of Reproductive traits

Table S1. Enrichment Analysis of candidate genes for Reproductive traits by GO

Trait	ontology	ID	Description	*p*-value
ALS6	MF	GO:0022824	transmitter-gated ion channel activity	6.46883E-05
	MF	GO:0022835	transmitter-gated channel activity	6.46883E-05
	MF	GO:0030594	neurotransmitter receptor activity	0.00011605
	MF	GO:0005230	extracellular ligand-gated ion channel activity	0.000164267
	MF	GO:0015276	ligand-gated ion channel activity	0.000498167
	MF	GO:0022834	ligand-gated channel activity	0.000498167
	MF	GO:0015318	inorganic molecular entity transmembrane transporter activity	0.000895914
	MF	GO:0015075	ion transmembrane transporter activity	0.001337601
	MF	GO:0022836	gated channel activity	0.003148918
	MF	GO:0005216	ion channel activity	0.005171508
	MF	GO:0015299	solute:proton antiporter activity	0.005414759
	MF	GO:0008066	glutamate receptor activity	0.005954942
	MF	GO:0015267	channel activity	0.006464157
	MF	GO:0022803	passive transmembrane transporter activity	0.006464157
	MF	GO:1904315	transmitter-gated ion channel activity involved in regulation of postsynaptic membrane potential	0.008113328
	MF	GO:0015491	cation:cation antiporter activity	0.008652338
	MF	GO:0099529	neurotransmitter receptor activity involved in regulation of postsynaptic membrane potential	0.008652338
	MF	GO:0015298	solute:cation antiporter activity	0.009191114
	MF	GO:0019213	deacetylase activity	0.009191114
	MF	GO:0005231	excitatory extracellular ligand-gated ion channel activity	0.009729656
	MF	GO:0016597	amino acid binding	0.009729656
	MF	GO:0098960	postsynaptic neurotransmitter receptor activity	0.012418853
	MF	GO:0015297	antiporter activity	0.01617391
	MF	GO:0005254	chloride channel activity	0.022584571
	MF	GO:0008146	sulfotransferase activity	0.02311728
	MF	GO:0043177	organic acid binding	0.023649757
	MF	GO:0005253	anion channel activity	0.026839747
	MF	GO:0015108	chloride transmembrane transporter activity	0.0273706
	MF	GO:0016782	transferase activity, transferring sulfur-containing groups	0.02896177

MF	GO:0015081	sodium ion transmembrane transporter activity	0.03108009
MF	GO:0031406	carboxylic acid binding	0.03372279
MF	GO:0052689	carboxylic ester hydrolase activity	0.03635972
MF	GO:0015078	proton transmembrane transporter activity	0.037939112
MF	GO:0015103	inorganic anion transmembrane transporter activity	0.041091685
MF	GO:0022853	active ion transmembrane transporter activity	0.048415555
CC	GO:0045211	postsynaptic membrane	0.00053967
CC	GO:0097060	synaptic membrane	0.000983841
CC	GO:0098794	postsynapse	0.00288054
CC	GO:0098982	GABA-ergic synapse	0.010528333
CC	GO:0034707	chloride channel complex	0.013520082
CC	GO:0098590	plasma membrane region	0.01470527
CC	GO:0045202	synapse	0.017444587
BP	GO:0001941	postsynaptic membrane organization	0.004823627
BP	GO:0015012	heparan sulfate proteoglycan biosynthetic process	0.006267306
BP	GO:0007340	acrosome reaction	0.006748184
BP	GO:0007628	adult walking behavior	0.006748184
BP	GO:0090659	walking behavior	0.007228886
BP	GO:0043113	receptor clustering	0.007709414
BP	GO:0030201	heparan sulfate proteoglycan metabolic process	0.008189768
BP	GO:0051668	localization within membrane	0.010109439
BP	GO:0007218	neuropeptide signaling pathway	0.012505107
BP	GO:0033692	cellular polysaccharide biosynthetic process	0.012505107
BP	GO:0006024	glycosaminoglycan biosynthetic process	0.013940419
BP	GO:0030166	proteoglycan biosynthetic process	0.014418509
BP	GO:0000271	polysaccharide biosynthetic process	0.014896424
BP	GO:0006023	aminoglycan biosynthetic process	0.015851734
BP	GO:0034637	cellular carbohydrate biosynthetic process	0.016329128
BP	GO:1903510	mucopolysaccharide metabolic process	0.016806349
BP	GO:0006885	regulation of pH	0.017283396
BP	GO:0008344	adult locomotory behavior	0.018236969
BP	GO:0099173	postsynapse organization	0.018713495
BP	GO:0006029	proteoglycan metabolic process	0.019666027
BP	GO:0044264	cellular polysaccharide metabolic process	0.02156901
BP	GO:0007338	single fertilization	0.022044323
BP	GO:0050905	neuromuscular process	0.022044323
BP	GO:0055067	monovalent inorganic cation homeostasis	0.022519462
BP	GO:0030203	glycosaminoglycan metabolic process	0.022994428
BP	GO:0005976	polysaccharide metabolic process	0.023943842
BP	GO:0006022	aminoglycan metabolic process	0.025366664
BP	GO:0044272	sulfur compound biosynthetic process	0.025366664
BP	GO:0009566	fertilization	0.027734577
BP	GO:0007601	visual perception	0.028207641
BP	GO:0050953	sensory perception of light stimulus	0.029625798
BP	GO:0030534	adult behavior	0.031042403
BP	GO:0016051	carbohydrate biosynthetic process	0.038102174
BP	GO:0007626	locomotory behavior	0.041383499
BP	GO:0044262	cellular carbohydrate metabolic process	0.049317478

UD	MF	GO:0015662	ion transmembrane transporter activity, phosphorylative mechanism	0.00141059
	MF	GO:0019829	ATPase-coupled cation transmembrane transporter activity	0.002604167
	MF	GO:0042625	ATPase-coupled ion transmembrane transporter activity	0.002604167
	MF	GO:0015079	potassium ion transmembrane transporter activity	0.009982639
	MF	GO:0046873	metal ion transmembrane transporter activity	0.022677951
	MF	GO:0022890	inorganic cation transmembrane transporter activity	0.032335069
	MF	GO:0008324	cation transmembrane transporter activity	0.035698785
	MF	GO:0015318	inorganic molecular entity transmembrane transporter activity	0.045898438
TD	MF	GO:0098631	cell adhesion mediator activity	0.001085069
	MF	GO:0050839	cell adhesion molecule binding	0.008029514
	CC	GO:0099061	integral component of postsynaptic density membrane	0.001208094
	CC	GO:0099146	intrinsic component of postsynaptic density membrane	0.001409443
	CC	GO:0098839	postsynaptic density membrane	0.001610792
	CC	GO:0099060	integral component of postsynaptic specialization membrane	0.001912816
	CC	GO:0098948	intrinsic component of postsynaptic specialization membrane	0.002114165
	CC	GO:0099634	postsynaptic specialization membrane	0.002416188
	CC	GO:0099055	integral component of postsynaptic membrane	0.002919561
	CC	GO:0098936	intrinsic component of postsynaptic membrane	0.003221585
	CC	GO:0098685	Schaffer collateral - CA1 synapse	0.003523608
	CC	GO:0099699	integral component of synaptic membrane	0.003926306
	CC	GO:0099240	intrinsic component of synaptic membrane	0.004329004
	CC	GO:0014069	postsynaptic density	0.006745193
	CC	GO:0032279	asymmetric synapse	0.006745193
	CC	GO:0045211	postsynaptic membrane	0.007449914
	CC	GO:0099572	postsynaptic specialization	0.007751938
	CC	GO:0098984	neuron to neuron synapse	0.007953287
	CC	GO:0097060	synaptic membrane	0.010067452
	CC	GO:0098978	glutamatergic synapse	0.011476895
	CC	GO:0098794	postsynapse	0.017316017
	CC	GO:0005887	integral component of plasma membrane	0.039766435
	CC	GO:0098590	plasma membrane region	0.039967784
	CC	GO:0031226	intrinsic component of plasma membrane	0.043088694
	CC	GO:0045202	synapse	0.043692741
	BP	GO:0050770	regulation of axonogenesis	0.005314652
	BP	GO:0098742	cell-cell adhesion via plasma-membrane adhesion molecules	0.009904578
	BP	GO:0050804	modulation of chemical synaptic transmission	0.014856867
	BP	GO:0099177	regulation of trans-synaptic signaling	0.014856867
	BP	GO:0050808	synapse organization	0.015460804
	BP	GO:0010975	regulation of neuron projection development	0.015943955
	BP	GO:0007409	axonogenesis	0.018359705
	BP	GO:0061564	axon development	0.020171518
	BP	GO:0048667	cell morphogenesis involved in neuron differentiation	0.023070419
	BP	GO:0007268	chemical synaptic transmission	0.023311994
	BP	GO:0098916	anterograde trans-synaptic signaling	0.023311994
	BP	GO:0099537	trans-synaptic signaling	0.023674357
	BP	GO:0099536	synaptic signaling	0.02451987
	BP	GO:0048812	neuron projection morphogenesis	0.025365382

	BP	GO:0120039	plasma membrane bounded cell projection morphogenesis	0.02596932
	BP	GO:0048858	cell projection morphogenesis	0.026090108
	BP	GO:0120035	regulation of plasma membrane bounded cell projection organization	0.026090108
	BP	GO:0031344	regulation of cell projection organization	0.02645247
	BP	GO:0032990	cell part morphogenesis	0.026694045
	BP	GO:0034330	cell junction organization	0.028264283
	BP	GO:0032989	cellular component morphogenesis	0.030800821
	BP	GO:0000904	cell morphogenesis involved in differentiation	0.032008697
	BP	GO:0098609	cell-cell adhesion	0.036477835
	BP	GO:0031175	neuron projection development	0.038772799
	BP	GO:0022603	regulation of anatomical structure morphogenesis	0.042879575
	BP	GO:0000902	cell morphogenesis	0.046865563
	BP	GO:0048666	neuron development	0.046986351
TL	BP	GO:0036002	pre-mRNA binding	0.001193576
	BP	GO:0033120	positive regulation of RNA splicing	0.001570238
	BP	GO:0000381	regulation of alternative mRNA splicing, via spliceosome	0.002174176
	BP	GO:0000380	alternative mRNA splicing, via spliceosome	0.002778113
	BP	GO:0048024	regulation of mRNA splicing, via spliceosome	0.003865201
	BP	GO:1903313	positive regulation of mRNA metabolic process	0.004227564
	BP	GO:0050684	regulation of mRNA processing	0.006039377
	BP	GO:0043484	regulation of RNA splicing	0.006522527
	BP	GO:1903311	regulation of mRNA metabolic process	0.011957966
	BP	GO:0000375	RNA splicing, via transesterification reactions	0.014132142
	BP	GO:0000377	RNA splicing, via transesterification reactions with bulged adenosine as nucleophile	0.014132142
	BP	GO:0000398	mRNA splicing, via spliceosome	0.014132142
	BP	GO:0008380	RNA splicing	0.020896244
	BP	GO:0006397	mRNA processing	0.024278294
	BP	GO:0016071	mRNA metabolic process	0.03478681
	BP	GO:0006396	RNA processing	0.046744776
	BP	GO:0010628	positive regulation of gene expression	0.046744776
SNT	MF	GO:0016755	transferase activity, transferring amino-acyl groups	0.002819344
	MF	GO:0019838	growth factor binding	0.013625882
	MF	GO:0004714	transmembrane receptor protein tyrosine kinase activity	0.014487861
	MF	GO:0004713	protein tyrosine kinase activity	0.016425938
	MF	GO:0019199	transmembrane receptor protein kinase activity	0.017501823
	MF	GO:0016746	transferase activity, transferring acyl groups	0.031007528
	CC	GO:0009986	cell surface	0.001921094
	CC	GO:0062023	collagen-containing extracellular matrix	0.020995108
	CC	GO:0005938	cell cortex	0.02515855
	CC	GO:0009897	external side of plasma membrane	0.025752362
	CC	GO:0030312	external encapsulating structure	0.03698902
	CC	GO:0031012	extracellular matrix	0.03698902
	CC	GO:0098552	side of membrane	0.046969343
	BP	GO:0032633	interleukin-4 production	0.004342575
	BP	GO:0032673	regulation of interleukin-4 production	0.004342575
	BP	GO:0032733	positive regulation of interleukin-10 production	0.005065113
	BP	GO:0032743	positive regulation of interleukin-2 production	0.005065113

BP	GO:0043372	positive regulation of CD4-positive, alpha-beta T cell differentiation	0.005065113
BP	GO:2000516	positive regulation of CD4-positive, alpha-beta T cell activation	0.005065113
BP	GO:0010837	regulation of keratinocyte proliferation	0.005787302
BP	GO:0010518	positive regulation of phospholipase activity	0.006148265
BP	GO:0046638	positive regulation of alpha-beta T cell differentiation	0.006509141
BP	GO:0010517	regulation of phospholipase activity	0.006869929
BP	GO:0046635	positive regulation of alpha-beta T cell activation	0.006869929
BP	GO:0060193	positive regulation of lipase activity	0.006869929
BP	GO:0032613	interleukin-10 production	0.00723063
BP	GO:0032653	regulation of interleukin-10 production	0.00723063
BP	GO:0043616	keratinocyte proliferation	0.007591244
BP	GO:0043370	regulation of CD4-positive, alpha-beta T cell differentiation	0.008312209
BP	GO:0032623	interleukin-2 production	0.009393002
BP	GO:0032663	regulation of interleukin-2 production	0.009393002
BP	GO:0046637	regulation of alpha-beta T cell differentiation	0.009393002
BP	GO:0060191	regulation of lipase activity	0.009393002
BP	GO:0048701	embryonic cranial skeleton morphogenesis	0.009753092
BP	GO:2000514	regulation of CD4-positive, alpha-beta T cell activation	0.010113094
BP	GO:0045582	positive regulation of T cell differentiation	0.012630669
BP	GO:0046634	regulation of alpha-beta T cell activation	0.012630669
BP	GO:0043367	CD4-positive, alpha-beta T cell differentiation	0.013708322
BP	GO:1904888	cranial skeletal system development	0.014067365
BP	GO:0045621	positive regulation of lymphocyte differentiation	0.015143972
BP	GO:0035710	CD4-positive, alpha-beta T cell activation	0.016219794
BP	GO:0046632	alpha-beta T cell differentiation	0.017653006
BP	GO:0048704	embryonic skeletal system morphogenesis	0.020157775
BP	GO:0045580	regulation of T cell differentiation	0.021229942
BP	GO:0050680	negative regulation of epithelial cell proliferation	0.021587158
BP	GO:0046631	alpha-beta T cell activation	0.022658282
BP	GO:0045619	regulation of lymphocyte differentiation	0.025866965
BP	GO:0048706	embryonic skeletal system development	0.02657905
BP	GO:1902107	positive regulation of leukocyte differentiation	0.026934963
BP	GO:1903708	positive regulation of hemopoiesis	0.026934963
BP	GO:0050870	positive regulation of T cell activation	0.02800218
BP	GO:1903039	positive regulation of leukocyte cell-cell adhesion	0.030844277
BP	GO:0046777	protein autophosphorylation	0.033326563
BP	GO:0022409	positive regulation of cell-cell adhesion	0.036158267
BP	GO:0051251	positive regulation of lymphocyte activation	0.03651184
BP	GO:0001818	negative regulation of cytokine production	0.039337317
BP	GO:0030217	T cell differentiation	0.040395445
BP	GO:0002696	positive regulation of leukocyte activation	0.043565166
BP	GO:0050863	regulation of T cell activation	0.043565166
BP	GO:1903037	regulation of leukocyte cell-cell adhesion	0.043565166
BP	GO:0050867	positive regulation of cell activation	0.043916926
BP	GO:0048705	skeletal system morphogenesis	0.045674431
BP	GO:1902105	regulation of leukocyte differentiation	0.045674431

Table S2. Enrichment Analysis of KEGG Pathway of candidate genes for Reproductive traits

Trait	ID	Description	*p*-value
ALS6	chx00534	Glycosaminoglycan biosynthesis - heparan sulfate / heparin	0.013504191
	chx04080	Neuroactive ligand-receptor interaction	0.015078267
	chx05033	Nicotine addiction	0.022425641
	chx05030	Cocaine addiction	0.027968689
	chx04730	Long-term depression	0.033486563
	chx04720	Long-term potentiation	0.036785241
	chx05031	Amphetamine addiction	0.038979351
UD	chx04964	Proximal tubule bicarbonate reclamation	0.00260181
	chx04960	Aldosterone-regulated sodium reabsorption	0.004298643
	chx04973	Carbohydrate digestion and absorption	0.005316742
	chx04961	Endocrine and other factor-regulated calcium reabsorption	0.005882353
	chx04978	Mineral absorption	0.00678733
	chx04918	Thyroid hormone synthesis	0.008710407
	chx04971	Gastric acid secretion	0.008936652
	chx04911	Insulin secretion	0.009841629
	chx04260	Cardiac muscle contraction	0.010294118
	chx04970	Salivary secretion	0.010633484
	chx04925	Aldosterone synthesis and secretion	0.010746606
	chx04972	Pancreatic secretion	0.011877828
	chx04976	Bile secretion	0.012330317
	chx04919	Thyroid hormone signaling pathway	0.013800905
	chx04974	Protein digestion and absorption	0.016628959
	chx04261	Adrenergic signaling in cardiomyocytes	0.017081448
	chx04022	cGMP-PKG signaling pathway	0.019004525
	chx04024	cAMP signaling pathway	0.026696833
TD	chx04514	Cell adhesion molecules	0.018099548
	chx04360	Axon guidance	0.020361991
TL	chx03060	Protein export	0.002714932
SNT	chx05230	Central carbon metabolism in cancer	0.007692308
	chx01521	EGFR tyrosine kinase inhibitor resistance	0.009049774
	chx05215	Prostate cancer	0.010972851
	chx04550	Signaling pathways regulating pluripotency of stem cells	0.016176471
	chx05226	Gastric cancer	0.01719457
	chx04015	Rap1 signaling pathway	0.023868778
	chx04810	Regulation of actin cytoskeleton	0.024886878
	chx04014	Ras signaling pathway	0.02658371
	chx04020	Calcium signaling pathway	0.026923077
	chx04144	Endocytosis	0.028959276
	chx04010	MAPK signaling pathway	0.033371041
	chx04151	PI3K-Akt signaling pathway	0.041742081
